# Chicken Gut Microbiota: Importance and Detection Technology

**DOI:** 10.3389/fvets.2018.00254

**Published:** 2018-10-23

**Authors:** Yue Shang, Sanjay Kumar, Brian Oakley, Woo Kyun Kim

**Affiliations:** ^1^St. Boniface Hospital Research Centre, Winnipeg, MB, Canada; ^2^Department of Animal Science, University of Manitoba, Winnipeg, MB, Canada; ^3^Department of Poultry Science, University of Georgia, Athens, GA, United States; ^4^College of Veterinary Medicine, Western University of Health Sciences, Pomona, CA, United States

**Keywords:** chicken, gut function, microbiome, prebiotics, DNA sequencing

## Abstract

Sustainable poultry meat and egg production is important to provide safe and quality protein sources in human nutrition worldwide. The gastrointestinal (GI) tract of chickens harbor a diverse and complex microbiota that plays a vital role in digestion and absorption of nutrients, immune system development and pathogen exclusion. However, the integrity, functionality, and health of the chicken gut depends on many factors including the environment, feed, and the GI microbiota. The symbiotic interactions between host and microbe is fundamental to poultry health and production. The diversity of the chicken GI microbiota is largely influenced by the age of the birds, location in the digestive tract and diet. Until recently, research on the poultry GI microbiota relied on conventional microbiological techniques that can only culture a small proportion of the complex community comprising the GI microbiota. 16S rRNA based next generation sequencing is a powerful tool to investigate the biological and ecological roles of the GI microbiota in chicken. Although several challenges remain in understanding the chicken GI microbiome, optimizing the taxonomic composition and biochemical functions of the GI microbiome is an attainable goal in the post-genomic era. This article reviews the current knowledge on the chicken GI function and factors that influence the diversity of gut microbiota. Further, this review compares past and current approaches that are used in chicken GI microbiota research. A better understanding of the chicken gut function and microbiology will provide us new opportunities for the improvement of poultry health and production.

## Introduction

The integrity of the gastrointestinal tract (GIT) and the gut microbial community play vital roles in nutrition absorption, development of immunity, and disease resistance. Alterations in the GIT microbial community may have adverse effects on feed efficiency, productivity, and health of chickens (1–3). Understanding the roles of the chicken GI microbiota and understanding the current methods used in microbiome research is essential for improving the poultry GI microbiome. Historically, selective culture-based techniques have been used to identify and characterize the microbial diversity of the avian gut. In the last decade, the use of bacterial 16S ribosomal RNA (rRNA) gene sequencing has dramatically improved our understanding of the composition and diversity of the chicken GI microbiota. Modern high-throughput sequencing approaches are capable of rapidly obtaining a complete census of a bacterial community and are a powerful tool that has led to important new insights into the biological and ecological roles of the GI microbiota. This review aims to summarize avian gut function as well as factors that influence the diversity of the chicken GI microbiota. Furthermore, we have also compared and reviewed past and current approaches used in chicken gut microbiological research.

## The role of chicken gastrointestinal microbiota

The gastrointestinal compartments of chickens are densely populated with complex microbial communities (Bacteria, fungi, Archaea, protozoa, and virus) that are dominated by Bacteria (4). The interactions between the host and the chicken GI bacterial microbiome have been extensively studied and reviewed by many research groups (5–9) and are now considered to play important roles in bird nutrition, physiology and gut development (10, 11).

The gut microbiota can form a protective barrier by attaching to the epithelial walls of the enterocyte and thus reduce the opportunity for the colonization of pathogenic bacteria (12). These bacteria produces vitamins (e.g., vitamin K and vitamin B groups), short chain fatty acids (acetic acid, butyric acid and propionic acid), organic acids (e.g., lactic acid) and antimicrobial compounds (e.g., bacteriocins), lower triglyceride, and induce non-pathogenic immune responses, which provide both nutrition and protection for the animal (2, 12–14). On the other hand, the GI microbiome can also be a source of bacterial pathogens such as *Salmonella* and *Campylobacter* which can disseminate to humans or act as a pool for antibiotic resistance and transmission and therefore may pose a serious threat to public health (5, 8, 15).

A normal gut microbial community has benefits and costs to the host (1, 13). The primary benefits that are provided by commensal microbiota are competitive exclusion of pathogens or non-indigenous microbes (13), immune stimulation and programming, and contributions to host nutrition. Earlier reports have established that conventionally raised animals are far less susceptible to pathogens when compared with germ-free animals (16). Furthermore, commensal microbiota can stimulate the development of immune system including the mucus layer, epithelial monolayer, the intestinal immune cells (e.g., cytotoxic and helper T cells, immunoglobulin producing cells and phagocytic cells), and the lamina propria (13, 17, 18). These tissues build barriers between the host and the microbes and combat undesirable gut microorganisms. In the distal gut (i.e., ceca and colon), the microbiota also produces energy and nutrients such as vitamins, amino acids, and short chain fatty acids (SCFA) from the undigested feed, which eventually become available for the host (1, 13). These SCFA have bacteriostatic properties that are capable of eliminating foodborne pathogens, such as *Salmonella* spp. (19). The SCFA are also a source of energy to the animals and can further stimulate gut epithelial cell proliferation, thus increasing the gastrointestinal absorption surface (13). It has also been established that SCFA production lowers the pH of colon, which inhibits conversion of bile to secondary bile products (20). In addition, gut microbiota also contributes to metabolism of host nitrogenous compounds. For example, cecal bacteria can convert uric acid to ammonia, which is subsequently absorbed by the bird and further used to produce amino-acids such as glutamine (21). Furthermore, some of the nitrogen from the diet gets incorporated into bacterial cellular protein and therefore, bacteria themselves can be a source of proteins/amino-acids (22).

In contrast, commensal microbiota also incurs cost to the host. In the proximal gut (gizzard and small intestine), microbes compete with the host for energy and protein. In both the proximal and distal gut, microbes produce toxic metabolites (e.g., amino acid catabolites) and catabolize bile acids, which may depress growth and decrease fat digestibility of the birds, respectively (1). In the presence of microbiota, the gut mucus layer increases mucin secretion and epithelial cell turnover rate, thereby keeping the GI tract lubricated while preventing microorganisms from invading intestinal epithelial cells of the host. The intestinal immune system is also more developed and secretes IgA, which specifically binds to bacterial epitopes, helps in regulating bacterial composition in the gut (23, 24). While generally beneficial, these processes do increase the demand for energy and protein from the host and therefore have an influence on the growth performance of the birds.

An imbalanced gut microbiota is often referred to as dysbiosis. Dysbiosis can been defined as qualitative and/or quantitative imbalance of normal microbiota in the small intestine, which may lead to a sequential reaction in the GIT, including reduced intestinal barrier function (e.g., thinning of intestinal wall) and poor nutrient digestibility, and therefore, increasing the risk of bacterial translocation and inflammatory responses (25). Both non-infectious and infectious stressors can lead to dysbacteriosis. The non-infectious factors include environmental stressors, nutritional imbalances, dietary changes, mycotoxins, poor management, enzymatic dysfunction, or host genetics (25). Infectious factors include viral or bacterial challenge, coccidiosis, or toxic metabolites produced by harmful microorganisms such as *Clostridium perfringens*.

The gastrointestinal microbiota can further be classified as the luminal microbiota and the mucosal microbiota (2). The composition of the luminal microbiota is determined by available nutrients, presence of antimicrobial substance and the feed passage rate. The composition of the mucosal-attached microbiota is affected by several host factors, such as expression of specific adhesion sites on the enterocyte membrane, secretion of secretory immunoglobulins, and mucus production rate. The luminal microbiota and the mucosal-associated microbiota of course also influence each other (2) and therefore, it is important to recognize that diet can alter both luminal and mucosal-attached microbiota to influence gut health. To our knowledge, there is no study to date which has compared the taxonomic composition or metabolic functions of these two microbial habitats. However, it would be interesting to study and analyse the variations between the bacterial communities of the mucosa and lumen throughout the different GI sections. Furthermore, studying the mucosal-associated bacterial community will be important to understand the host mucosal responses as any alterations in mucosal immunity may have serious implications on bird's health (26).

## The diversity of chicken gut microbiota

The GI tract of the chicken harbors a diverse bacterial community in which each bacterium is adapted to its own ecological niche and synergistically lives with other bacterial species in the same community. The composition and function of these communities has been shown to vary depending on the age of the birds, location in the GI tract and on the dietary components (6, 18, 27–1^29^).

## Bird age

The age of the birds is one of the most important factors that influences GI bacterial composition, cell density, and metabolic function. Significant changes in the taxonomic composition of gut microbiota have been studied using both DNA finger-printing (30) and high-throughput sequencing approaches (31) and are well-reviewed by many research groups (28, 32–34). Ballou et al. (35) and our recently published data (5) indicates that 1 day post-hatch broiler chicks already have a microbial community in their GIT. There are also successional changes in the composition of the GIT microbiome, due to the replacement and establishment of more stable bacterial taxa, as the bird advances in age (30, 36). Lu et al. (30) discovered that the GIT of chicken at 3 days of age contained *L. delbrueckii, C. perfringens* and *Campylobacter coli*, whereas from 7 to 21 days of age, *L. acidophilus, Enterococcus*, and *Streptococcus* were more common. At 28 and 49 days of age, the GI tract contains *L. crispatus*, but the composition is significantly different from other ages (30). In other work, successional changes in the gut microbial community measured with HT-NGS technology has shown that the relative abundance of *Clostridium* was higher as the bird aged, whereas lactobacilli was low throughout the growth cycle. This variability in results may be due to sample types (feces vs. cecum), and/or conventional microbiological and molecular methods that have limited coverage and accuracy compared to high-throughput NGS platforms which offer higher coverage and depth in determining microbial community. High-throughput sequencing technologies, such as targeted amplicon sequencing and shotgun metagenomic sequencing, have become more common to analyze the gut microbial composition and functions throughout the life span of broilers, but we are still at initial stage of analyses and there is a breach in knowledge regarding host morphological development, and functional properties of the gut microbiome as the bird ages.

## Gastrointestinal tract

The GI tract of the chicken includes the crop, proventriculus, gizzard, duodenum, jejunum, ileum, caeca, large intestine, and cloaca (32). Each GI tract section has different metabolic functions that shape the microbial community (Table 1), and therefore it is important to consider sampling location and study design. The chicken crop harbors 10^8^ to 10^9^ cfu/g bacteria, which is usually dominated by lactobacilli (28, 37). However, large variations in microbial composition among individual broilers fed on the similar diet has been observed by Choi et al. (44) due to difference in time between feeding and sampling. In the gizzard, the concentration of bacteria is similar to the crop, but bacterial fermentation activities are low mainly because of the low pH. The majority of bacteria in the gizzard are lactobacilli, enterococci, lactose-negative enterobacteria, and coliform bacteria (28). Among the small intestinal segments, the bacterial density is the lowest in the duodenum due to short passage time and a dilution of digesta by secreted bile (45). The duodenal bacterial community mainly consists of clostridia, streptococci, enterobacteria, and lactobacilli (46). Ileum microbiota have been studied the most among the small intestine segments. Lu et al. (30) assessed the ileal bacterial community by examining 16S rRNA gene sequences and found *Lactobacillus* as the major group (70%) followed by members of the family *Clostridiaceae* (11%), *Streptococcus* (6.5%) and *Enterococcus* (6.5%) (30). In corroboration, our recent article also showed lactobacilli as the predominant genus in the ileum (5). Compared to the ileum, the cecum harbors a more diverse, rich and stable microbial community including anaerobes (47, 48). Oakley et al. (18) have documented significant changes in cecal microbial communities from day of hatch to 6 weeks of age in commercial broilers (18, 27) and also significant differences in cecal vs. fecal samples from a single individual (27). Typically, richness and diversity in the cecum increase during these 6 weeks, and the taxonomic composition of the community quickly shifts from Proteobacteria, Bacteroides, and Firmicutes, to almost entirely Firmicutes by 3 weeks of age (18, 27). However, Kumar et al. (5) found that Firmicutes were the most abundant phylum in both ceca and ileum at all the ages (day 0 to day 42) except d 42 in the ceca where Bacteroidetes were abundant. The differences in bacterial composition can be expected due to differences in the nucleic acid extraction protocol, primers, sequencing approach, environmental factors, dietary treatment/ composition, breed, and geographical conditions. In addition to sample types, an adequate sample size is also needed for a proper study design. Higher individual variation in sample types (crop samples) results in higher sample size compared to cecal samples to find the potential differences (49).

**Table 1:** Spatial distribution of most common and abundant bacterial taxa (phylum, order (o), family (f), genus) in the gastro-intestinal tract of chickens irrespective of age, diet and technique differences.

**GIT location (per g of content)**	**Bacterial phyla**	**Bacteria genera**	**Techniques used**	**References**
Crop (10^8^−10^9^/ g)	Firmicutes	*Lactobacillus*	16 S rDNA sequencing and cloning	(37)
	Actinobacteria	*Bifidobacterium*		
	Proteobacteria	*Enterobacter*		
Gizzard (10^7^−10^8^/ g)	Firmicutes	*Lactobacillus, Enterococcus*		
Small Intestine (most of the studies are conducted in Ileum; 10^8^−10^9^/ g)	Firmicutes/ Low G+C, Gram positive bacteria	Enterococcaceae (f.), *Enterococcus*, Clostridiaceae (f.), *Clostridium*, Lactobacillacae (f.) *Lactobacillus, Candidatus Arthomitus, Weisella, Ruminococcus, Eubacterium, Bacillus*, Stapylococcaceae (f.), *Staphylococcus, Streptococcus, Turicibacter, Methylobacterium*	Finger printing: T-RFLP, 16S rRNA qPCR, Cloning and sequencing and Next Generation Sequencing	(5, 30, 38–40)
	Cytophaga/ Flexibacter/ Bacteroides/ High G+C, Gram positive bacteria	Bacteroidaceae (f.), *Bacteroidetes, Flavibacterium, Fusobacterium, Bifidobacterium*		
	Protobacteria	*Ochrobaterium, Alcaligenes, Escherichia, Campylobacter, Hafnia, Shigella,*		
	Actinobacteria/ Cyanobacteria	*Corynebacterium*		
Caeca (10^10^−10^11^/ g)	Methanogenic Archaea (0.81%)	*Methanobrevibacter, Methanobacterium, Methanothermobacter, Methanosphaera, Methanopyrus, Methanothermus, Methanococc*	Finger printing: T-RFLP, 16S rRNA qPCR, Cloning and sequencing and Next Generation Sequencing	(5, 30, 38, 39, 41–43)
	Firmicutes/ Low G+C, Gram positive bacteria (44–56%)	*Anaerotruncus*, Ruminococcaceae (f) *Ruminoccoccus, Faecalibacterium, Lachnospirceae, Bacillus, Streptococcus*, Clostridiales (o), *Clostridium, Megamonas, Lactobacillus, Enterococcus, Weisella, Eubacterium, Staphylococcus, Streptococcus,*		
	Bacteroides/ Cytophaga/ Flexibacter/ High G+C, Gram positive bacteria (23–46%)	Rikenellaceae (f), *Bacteroidetes, Alistipes, Fusobacterium, Bifidobacterium, Flavibacterium, Odoribacter,*		
	Actinobacteria	*Corynebacterium*		
	Proteobacteria (1–16%)	*Ochrobaterium, Alcaligenes, Escherichia, Campylobacter*		
Large Intestine	Firmicutes	*Lactobacillus*	16 S rDNA sequencing and cloning	(37)
	Proteobacteria	*Escherichia*		

Feed processing approaches, feed components and additives are also known to have an effect on the gut microbial community. Knarreborg et al. (50) stated that mash feed lowers the number of *Enterococcus* spp. and coliforms but increases *Lactobacillus* spp. and *C. perfringens* in the broiler ileum, when compared to pellet feed (50). Corn favors low percent G + C clostridia, enterococci and lactobacilli, whereas wheat favors higher percent G + C bifidobacteria (29). Kumar et al. (5) reported low abundance in Firmicutes and high abundance in Bacteroidetes from day 0 to day 42 as birds were shifted from starter diet to finisher diet and argued that members of the phylum Bacteroidetes are vital for fermenting starch to simple sugars. Furthermore, feed supplementation, such as fermentable sugars (prebiotics), can also have an impact on the composition and diversity of chicken gut microbiota.

## Prebiotics

The use of prebiotics as dietary modulators has been shown to have positive effects on some bacterial taxa in the colon (51). For example, Fructooligosaccharides (FOS) and Galactooligosaccharides (GOS) increased the population of *Bifidobacterium* and *Lactobacillus* (52, 53). *In vitro* studies have shown that fecal slurries which were incubated with oligofructose and inulin exhibited an increase in bifidobacteria populations in the human large intestine, whereas potential pathogens such as *Escherichia coli* and *Clostridium* spp. were maintained at lower levels (54). The majority of bifidobacteria strains (e.g., *B. fiagilk, B. thetaiotaomicron*, B*. vulgatus, B. dktasonk*, and *B. ovatus*) except *B. bifidum*, can utilize FOS as a growth and fermentation promoter (55). These bacteria secrete ß-fructosidase enzyme that can readily degrade and ferment FOS. However, microorganisms such as *E. coli* and *C. perfringens* are not able to exploit FOS as a fermentative carbohydrate source. Rats that were fed dietary FOS have shown a temporary boost in lactic acid-producing bacteria and a long-term elevation in cecal butyric acid (56). Dietary inclusion of FOS reduced *C. perfringens* and *E. coli* populations and increased the diversity of *Lactobacillus* in the broiler GIT (57). Patterson et al. (58) assessed the effects of thermal ketoses oligosaccharides on cecal microbial populations of broiler chickens. The results showed that cecal bifidobacteria and lactobacilli concentrations were increased 24-fold and 7-fold, respectively, in ketoses supplemented diet compared to controls. Another type of prebiotics, mannooligosaccharides (MOS), are proposed to have different mechanisms of action (58). They can (1) bind to potential pathogenic Gram-negative bacteria (e.g., *E. coli* and *Salmonella*) which possess type-1 fimbriae (mannose-sensitive lectin), to prevent and dislocate the pathogens from attaching to the gut wall, (2) have immune modulatory effects based on the antigenicity features of mannan and glucan components, (3) modulate intestinal morphology, and (4) enhance the expression of mucin and reduce enterocyte turnover rate (59). The effects of prebiotics on lower GI tract include: (1) serving as food and fermentation sources for cecal and colonic microbiota, (2) production of fermentation end products (e.g., SCFAs), (3) stimulation of saccharolytic fermentation, (4) acidification of the large intestine content, (5) hyperplasia of the cecal and colonic epithelium, (6) stimulation of colonic hormonal peptides secretion, and (7) acceleration of ceco-anal transit (51).

Other than age, GIT location, and prebiotics, breed and sex of the bird can also have a large impact on the intestinal microbiota (34). In addition, it has been well-documented that environmental factors (biosecurity level, housing, litter, feed access, and climate) can also substantially influence the gut bacterial composition. Therefore, data interpretation and outcome of research largely depends on the study design. Best practices for research reporting include providing details regarding host and environmental factors that can enable researchers to do meta-analyses to better understand nutritional, microbiome, and environmental factors that can be modulated to improve bird performance and health.

## Discovery of chicken gut microbiota by molecular approaches

Classical culture-based methods have historically been widely used to study the chicken gut microbiota. However, these methods are highly selective to cultivable bacteria under specific conditions (60). A majority of bacteria remain uncultured (29). Over 30 years ago, the term “the great plate count anomaly” was coined to reflect laboratory calculations that a very small minority (0.1–1%) of microbial taxa present in a given sample could be cultured (61). Similarly, over 10 years ago, it was observed that of 52 microbial phyla recognized at the time, only half of them had even a single cultivated representative, supporting the description of an “uncultivated majority” (62). Therefore, the richness (number of species) and diversity (number of species weighted by their relative abundance) of intestinal bacteria have been underestimated, and our knowledge of gut microbiota remains incomplete (63).

The development of molecular biotechnology has offered new tools to study the composition, diversity, predicted function and interaction of gut microbiota in different sections of the GI tract. Currently, a variety of molecular techniques are available, each with different strengths and weaknesses. The sample capacity, applications and limitations of some of the most common molecular techniques that can be used to study chicken GI microbial ecology are listed in Table 2. Among these methods, high-throughput sequencing of 16S rRNA gene amplicons has quickly become the method of choice. Although this method had been widely used in other research fields, the first report utilizing high-throughput sequencing of 16S rRNA genes for studying the population of microbial communities and their interactions in the chicken gut was published in 2013 (64).

**Table 2:** 16S rRNA-based molecular approaches for studying microbial ecology in the chicken gut (64–67).

**Approach**	**Sample capacity**	**Applications**	**Challenges and confines**	**Advantage**
**SEQUENCING ANALYSIS TARGETED AMPLICONS**				
16S rDNA sequencing	Limited w/ Sanger sequencing. Non-limiting w/ next-gen sequencing	16S rRNA gene sequence, wide range identification of genus/ species/ strain, as database rich	Bias in DNA extraction and Primers, PCR amplification and numbers of clones, costly, laborious	Each clone represents single molecule of rDNA, Allows precise identification of a relatively small number of OTUs
Real-time PCR (RT-PCR)	Limited	Specific gene expression in targeted groups, high in sensitivity	Bias in DNA extraction and RT-PCR, costly	
**PROFILING APPROACHES**				
Fingerprinting DGGE^a^, TGGE^b^, TTGE^c^, T-RFLP^d^, and SSCP^e^	Good	Amplify common 16S rDNA sequences, diversity profiles within the targeted group, rapid, comparative	Bias in DNA extraction, primers, inter and intra laboratory reproducibility remains a major challenge. Provides relatively coarse taxonomic resolution, data usually is qualitative or semi-quantitative	Amplicons may be used from sequencing
**GENE QUANTIFICATION**				
FISH^6^	Limited	Enumeration of the bacterial population	Laborious at the species level	Sensitivity has been improved using fluorescent probes
**DNA MICROARRAY TECHNOLOGY**				
Diversity arrays	High	Diversity profiles, different gene expression levels	Laborious in development, costly	
DNA microarrays	High	Transcriptional fingerprint, comparative	Bias in nucleic acids extraction and their labeling, costly	

a *DGGE, denaturing gradient gel electrophoresis*;

b *TGGE, temperature gradient gel electrophoresis*;

c *TTGE, temporal temperature gradient gel electrophoresis*;

d *T-RFLP, terminal restriction fragment length polymorphism*;

e *SSCP, single strand conformation polymorphism; ^f^ FISH, fluorescence in situ hybridization*.

**Figure 1:**
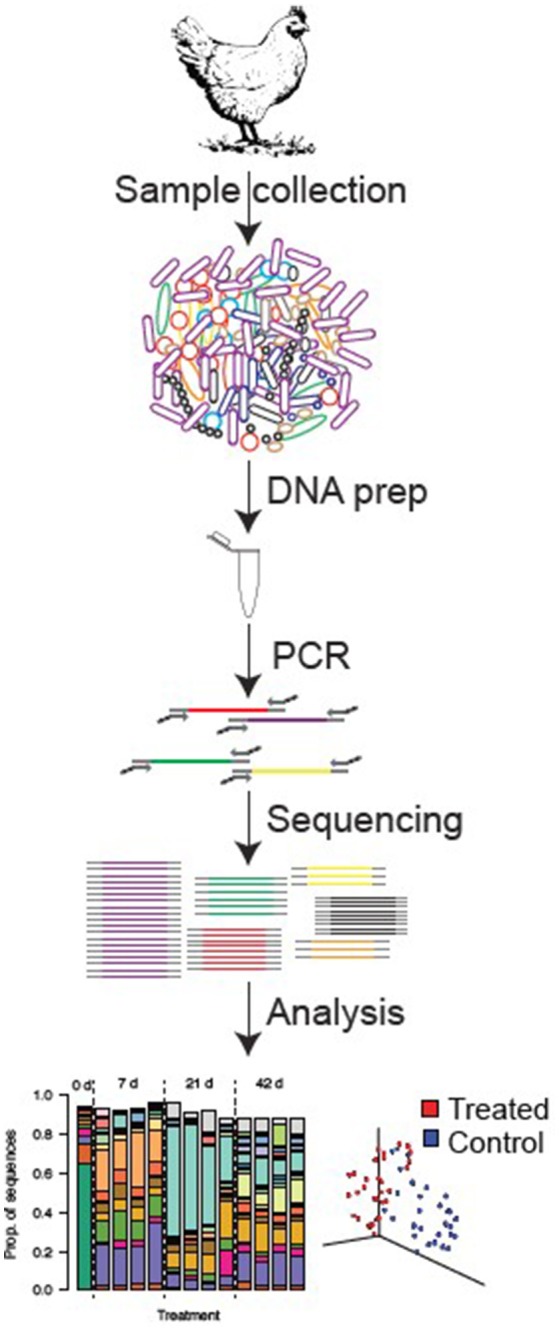
Standard procedure from sample collection to sequencing analysis in poultry gut.

The 16S rRNA molecule is a small subunit of the ribosome that possesses regions of sequence similarity that are highly conserved across all bacteria. To amplify these genes, microbial DNA is extracted from fecal or digesta samples, and broad-range primers, which target conserved regions of the 16S rRNA gene, are used for polymerase chain reaction (PCR) amplification (29). Sequencing of these amplified products (amplicons) can discriminate among bacteria, generally to the genus or species level (68, 65), and the relative abundance of each sequence reflects the relative abundance of that bacterium in the original sample. Thus, sequencing of 16S rRNA genes provides a true census of a bacterial community by defining the types of bacteria present in a sample and their relative abundances. Because of the high richness and diversity of intestinal bacterial communities, it has only been in the last few years that DNA sequencing technology has matured to the point where we can now completely census these complex communities. Beginning in 2008, technical advances in sequencing allowed for several orders of magnitude more sequences to be collected than was previously possible—in a single study the authors deposited as many 16S rRNA sequences in the GenBank database as had been generated historically up to that point (69). With these profound methodological advances and enormous new datasets, it is now possible to easily and accurately take a census of an intestinal sample to determine, for example, how the microbiome responds to different feed additives, husbandry conditions, or disease states (Figure 1).

High-throughput or next generation sequencing (NGS), is a powerful tool to investigate the biological and ecological role of gut microbiota (64). NGS has become a convenient, rapid, accurate and inexpensive method for genomic research (70, 66). Current NGS platforms offer high throughput, fast turn-around times, and low costs. Among these platforms the Illumina HiSeq and MiSeq instruments are two of the most frequently used systems in recent chicken gut microbiome and metagenomic research. Despite many advantages, these platforms suffer from limitations including short read assembly and high cost (71). Third-generation sequencing platforms such as single molecule real-time (SMRT) and nanopore sequencing require less time for DNA preparation (no PCR) and are cost effective (71). As these platforms continue to mature, their adoption will surely lead to new understanding of the poultry GI microbiome.

Following sequencing, bioinformatic analyses of sequence data requires open source platforms such as QIIME or mothur which utilize public databases (GreenGenes, Ribosomal Database Project and SILVA 72–76 to perform taxonomic assignment. Predictions of metabolic functions based on taxonomic identities from 16S rRNA gene sequences can be further obtained using algorithms such as PICRUSt and Tax4Fun 77, 78. To catalog the gene functions or analysis of individual genomes, metagenomic or metatranscriptomic approaches (in which genes or transcripts respectively are sequenced directly with no PCR) can be used to provide information on community diversity, structure and metabolic functions, or gene expression (79). Bioinformatic analyses of such datasets are more complex than 16S amplicon data and typically involve a sequence assembler such as Velvet (CLC workbench, Newbler version 3.0, Biospace) or MG-RAST. Bacterial taxa and functional groups can be assigned based on Basic Local Alignment Search Tool (BLAST), and gene functions may be analyzed using either Kyoto Encyclopedia of Genes and Genomes (KEGG) or Cluster of Orthologous genes (COG). In the chicken gut microbiome, metagenomics has been used to study the cecum functions, gut response to pathogen challenge, correlations between microbial response and performance parameters, comparison between fat and lean broiler lines, description on virulome, and antibiotic resistance genes (80). Some of the NGS based studies investigating chicken gut microbial community composition and functions in respect to the dietary responses/ antibiotic treatments are depicted in Table 3. However, it's difficult to compare all these studies because of variation in NGS platforms used, breed, sample type, sampling method etc. Therefore, a standard protocol is needed for studying the chicken gut microbial community, as available for human microbiome, in order to have comparable results. Currently most metagenomic approaches to studying the chicken GIT are still not affordable for most researchers or veterinarians.

**Table 3:** : Different omics approaches applied in understanding gut microbial community and functions.

**Omic approach**	**NGS platform**	**Research focus**	**Diet**	**Breed**	**Sample type**	**Sampling time**	**References**
Meta-proteomics		Correlation between metagenome and proteome of a healthy chicken	Attlee's non-medicated poultry feed	White Leghorn chickens	Feces	18 wesk	(81)
		Dietary effect of mineral phosphorus and microbial phytase on protein inventory of the microbiome	3 diets with P derived from plant source (BD-), 3 diets with P supplementation (BD+), BD- and BD+ supplemented with 0, 500 and 12,500 U/kg of phytase	Ross 308	Crop, ceca	25 day	(82)
Meta-genomics	454 pyrosequencing	Role of microbial community and functional gene content in caeca	Commercial chicken feed (Eagle milling)	Ross x Ross	Ceca	28 day	(41)
	454 pyrosequencing and shotgun metagenomics	Analyze effects of subtherapeutic doses of antimicrobials and anticoccidial on bacterial popoulation	Basal diet for 7 day followed by supplementation of monensin, monensin + virginiamycin or tylosin	Ross x Ross	Ceca	0,7,14,35 day	(7)
	MiSeq 2000	Deep microbial community profiling in the caeca and functional analysis	Wheat based diet with 5% maize (no antibiotics)	Ross x Ross	Ceca	42 day	(42)
	Shotgum metagenomics	Comparing fecal microbiome of low and high FCR brids	Growers diet	Broiler strain ‘MY’	Feces	49 day	(83)
	MiSeq 2000	Determining protein expression in the cecal microbiota in chickens of selected ages and in 7-day-old chickens inoculated with different cecal extracts on the day of hatching	Common mashed/granulated MINI feed	ISA Brown egg-laying hybrid	Ceca	Donor (1,3,16,28,42 week); Recipient (7 day old)	(84)
	HiSeq 2000	Metanalysis of antibiotic resistance genes and their co-occurrence with genetic elements	Commercial diet	NM	Feces	20, 80 day	(85)
	454 Genome Sequencer	Determine effect of diet on antibiotic resistance genes of gut microbiome	Basal diet with chlortetracycline and organic diet w/o antibiotic	Brown Leghorn	Feces	90 day	(9)
	HiSeq2000	Existence, diversity and abundance of antibiotic resistant genes	Commercial diet	NM	Feces	6 week broilers and 52 week laying hens	(86)
	MiSeq/ HiSeq4000	Metagenomic analysis for changes in bacterial community, antibiotic resistance genes in gut microbiota	Commercial diet with low and therapeutic dose level of chlortetracycline	NM^*^	Feces	0,5,10,20 day	(87)
16S rRNA targeted	454 pyrosequencing	Determine fecal microbiota subjected to repeated cycle of antimicrobial therapy	Basal diet with single cycle and repeated cycle of antibiotic therapy	Female Lohmann Brown layers	Feces	0,1,2,3,4,7,8,9,10,11,14,14,16,17,18,21,22 day	(48)
	MiSeq	Influence of genetic background of host on microbiome	Corn-soybean diet	NM	Feces	245 day females and males	(88)
	454 pyrosequencing	Investigate poultry-associated microbiome and food pathogens from farm to fork	Commercial diet supplemented with sub-therapeutic dose of antibiotic growth promoters	Ross x Hubbard	Feces, ceca, litter, carcass	6 week	(18)
	MiSeq	Effect of host genetic on microbiome and correlation with body weight	Corn-soybean	NM	Feces	258 d LW^$^ and HW males and females	(89)
	MiSeq	Effect of age o the gut microbial dynamics	Commercial broiler diet	Cobb 500	Ilea, ceca	7,14,21,42 d	(31)
	MiSeq v2 (500 cycle)	Investigate role of prebiotics on microbiome of pasture flock raised birds	Basal diet	NM	Ceca	8 weeks	(90)
	HiSeq 2000	Determine link between variation in fatness and gut microbiota	Commercial diet	NM	Feces	37 to 40 Week, from fat and lean chickens	(91)
	HiSeq 2000	Comparison of fat and lean chickens on gut microbiota	Commercial diet		Feces	35 weeks	(92)
	454 sequencing	Evaluate effect of diet and age on gut microbiota	Wheat-based diet, Maize-based diet or maize-based concentrates supplemented with 15% or 30% crimped kernel maize silage	Ross 308	Crop, gizzard, ilea, ceca	8,15,22,25,29,36 day	(93)
	MiSeq	Effect of antibiotic withdrawal from broiler feed on gut microbial community	Commercial diet with and without Bacitracin	Cobb 500	Ceca, ilea	0,7,14,22,35,42 day	(5)
	MiSeq600	Examine the effect of age, sample type, flock and successive flock cycles on consistency and predictability of the bacterial community	NM	Cobb 500	Ceca, ilea	7,14,21,28,35,42 day	(94)

* *NM, not mentioned*,

$ *LW, low weight and HW, high weight*.

To circumvent some of the confines of sequence-based analysis, proteomic methods have also recently been used to determine the metabolic and functional properties of the microbiome (81, 82). Transcriptomics measures gene transcription *in situ*, providing an accurate reflection of physiological functions even if utmost care is needed during sampling (71). Since there are limited culture collections for poultry strains, increase in bacterial cultures and proper cataloging of their biochemical and genetic properties will facilitate proteomics and other “omics” approaches.

## Conclusion

In recent years, significant progress has been made in understanding the taxonomic composition of the GI microbiome and its contributions to gut health. It is important for future studies to apply multi-omics approaches in order to increase our understanding of the role of the microbiome in nutrition, health, disease, and productivity. Progress in this field will help us to better understand how to manage the gut microbiota based on the environment, diet and physiology changes of the birds, and will further advance our understanding on the modification of microbiota-associated metabolic pathways, thus providing new opportunities for improving overall health of the poultry.

## Author contributions

YS and SK wrote this review manuscript. BO reviewed literature and the manuscript and provided critical suggestion and comments. WK decided a review topic, reviewed literature, and provided critical review and suggestion/comments.

### Conflict of interest statement

The authors declare that the research was conducted in the absence of any commercial or financial relationships that could be construed as a potential conflict of interest.
